# Text-Based vs. Graphical Information Formats in Sepsis Prevention and Early Detection: A Randomized Controlled Trial on Informed Choice

**DOI:** 10.3390/jcm11133659

**Published:** 2022-06-24

**Authors:** Luka Johanna Debbeler, Anne Pohrt, Carolin Fleischmann-Struzek, Daniel Schwarzkopf, Sebastian Born, Konrad Reinhart, Odette Wegwarth

**Affiliations:** 1 Center for Adaptive Rationality, Max Planck Institute for Human Development, Lentzeallee 94, 14195 Berlin, Germany; luka-johanna.debbeler@charite.de; 2 Heisenberg Chair for Medical Risk Literacy & Evidence-Based Decisions, Center for Anesthesiology & Intensive Care, Charité–Universitätsmedizin, Charitéplatz 1, 10117 Berlin, Germany; anne.pohrt@charite.de; 3 Center for Infectious Diseases and Infection Control, Jena University Hospital, Erlanger Allee 103, 07747 Jena, Germany; carolin.fleischmann@med.uni-jena.de (C.F.-S.); daniel.schwarzkopf@med.uni-jena.de (D.S.); sebastian.born@med.uni-jena.de (S.B.); konrad.reinhart@sepsis-stiftung.de (K.R.); 4 Center for Sepsis Control and Care, Jena University Hospital, Am Klinikum 1, 07747 Jena, Germany; 5 Center for Anesthesiology & Intensive Care, Charité University Medical Center Berlin, Charité–Universitätsmedizin, Charitéplatz 1, 10117 Berlin, Germany

**Keywords:** sepsis, informed choice, risk and health literacy, prevention, early detection, knowledge

## Abstract

Sepsis is associated with 11 million global deaths annually. Although serious consequences of sepsis can generally be avoided with prevention and early detection, research has not yet addressed the efficacy of evidence-based health information formats for different risk groups. This study examines whether two evidence-based health information formats—text based and graphical—differ in how well they foster informed choice and risk and health literacy and in how well they support different sepsis risk groups. Based on a systematic literature review, two one-page educative formats on sepsis prevention and early detection were designed—one text based and one graphical. A sample of 500 German participants was randomly shown one of the two formats; they were then assessed on whether they made informed choices and on their risk and health literacy. For both formats, >70% of participants made informed choices for sepsis prevention and >75% for early detection. Compared with the graphical format, the text-based format was associated with higher degrees of informed choice (*p* = 0.012, OR = 1.818) and risk and health literacy (*p* = 0.032, OR = 1.710). Both formats can foster informed choices and risk and health literacy on sepsis prevention and early detection, but the text-based format appears to be more effective.

## 1. Introduction

Every year, 11 million people worldwide die from sepsis, which is the primary cause of death from infection [1,2]. In Europe, incident sepsis cases in 2017 amounted to 2,430,000, with 545,000 deaths resulting; in Germany alone in 2016, there were 280,000 sepsis cases, leading to 55,000 deaths [1,3]. Survivors often face long-term functional, cognitive, or physical disabilities [4]. These numbers are particularly depressing given that many sepsis-associated deaths could be prevented with measures such as vaccinations, e.g., against influenza or pneumococcal infection, good wound hygiene, and timely detection and treatment [5,6,7,8,9]. Effectively addressing the threat of sepsis requires people to have a basic knowledge of potential causes and symptoms, as well as the benefits and harms of preventive measures, in order to make informed choices about potential courses of action [10]. However, studies on sepsis-associated awareness and knowledge in various countries have demonstrated considerable knowledge gaps [11,12,13]. For example, an international survey on public perceptions of and attitudes toward sepsis showed that 88% of participants from Italy, Spain, the United Kingdom, France, and the United States had never heard of sepsis [13]. In a German survey, 89% of participants over 60 reported that they had heard of sepsis, but they tended to underestimate the incidence and mortality rates of sepsis, and only 17% were aware that vaccination can help prevent sepsis [14].

One reason for the lack of awareness around sepsis might be that the available information on sepsis is not understood, in part due to incomplete or intransparent information formats. Many people, including patients who have to make decisions about their health, encounter difficulties in understanding medical information and risks [15,16,17,18,19,20]. However, certain presentation formats can help people understand numerical information [16,18,21,22,23]. For example, statistical formats such as absolute risk information have been shown to foster the comprehension of medical benefits and harms. In contrast, relative risk information misleads doctors and laypeople alike [16,24,25,26,27]. This is because relative numbers—in contrast to absolute numbers—hide the denominator they relate to, thereby providing incomplete and misleading risk information [16,28,29]. Furthermore, presenting health information in transparent graphical formats rather than in text-based formats can help people understand risks and make informed decisions [30]. For instance, graphical representations can increase the probability of preventive health behaviour and foster the recall of health information [30,31,32,33,34,35].

Nonetheless, studies show that when it comes to presentation formats, one size may not fit all: Different target groups (defined, e.g., by age, numeracy, or graph literacy) may require different forms of presentation to best improve their informed choices [19,34,36,37]. For instance, older patients tend to face more health risks and need to make more medical decisions; it is therefore necessary to tailor information formats to their specific needs.

The aim of the present study is to determine whether two evidence-based health information formats—text based and graphical—differ in how well they foster informed choice (primary endpoint), how well they foster risk and health literacy on sepsis (secondary endpoint), and how well they support different sepsis risk groups.

## 2. Materials and Methods

### 2.1. Study Overview

We employed an online randomized-controlled (text-based vs. graphical) trial (RCT). Participants were randomly assigned to either a text-based or a graphical health format at a 1:1 ratio. Participants were informed about the purpose of the study but were blind to the condition they were assigned to. This RCT is part of the SepsisWissen project funded by the Innovationfonds of the German Federal Joint Committee (01VSF19020). We registered the trial (Risk communication on sepsis early detection and prevention; RICOSEP) at the German Clinical Trial Register (DRKS00024850) and adhered to the CONSORT checklist. The original registration was amended in two ways after a soft launch with 30 recruited participants. While initially a pre–post design was planned, we had to decide against that after screening the data from the soft launch. Assessing the outcome measures prior to the intervention with the prequestionnaire directed participants’ attention towards the aspects addressed in the prequestionnaire. Thus, as they read through the risk information, a strong attentional bias was created which would not exist in a real-world setting and might have created a ceiling effect of measured endpoints regardless of format, which would have limited the validity of the trial. Furthermore, an additional item was included in the study that queried which 3 of 15 pieces of information about sepsis participants thought were the most important to know.

Recruitment was restarted for the adapted study design. The data from the 30 soft-launch participants were not included in the main analyses. However, some highlights which might provide exploratory insights are outlined in this paper and in the Supplementary Materials (Table S3).

### 2.2. Procedure

Participants gave informed consent prior to the study. Participants were screened according to preset criteria for age, pre-existing conditions (see Supplementary Materials, Table S1), and education. Either the text-based or the graphical health format was shown. Participants could spend as much time as they wanted to read the educative material, but based on pre-testing, a time minimum was set for 2.5 min, after which participants could proceed to the main survey.

### 2.3. Sample Size

In order for one information format to be considered superior, we required the conservative difference in informed choice to be at least 15 percentage points. The rationale for this benchmark was based on effects from survey studies comparing currently available standard information to either of the risk formats we used in our trial [38,39,40]. Taking these postulated differences into account (52% vs. 37% informed choice), planning for a chi-square test and aiming for a power of 90% at an α level of 5% (two-tailed), we needed 242 participants for each intervention arm (nQuery 7.0).

### 2.4. Participant Characteristics

Altogether, 500 people at higher risk for sepsis—that is, aged ≥ 60 years and/or presenting with pre-existing conditions such as cancer or chronic diseases—participated. Recruitment was undertaken by the market research institute IPSOS Health (Nuremberg, Germany). IPSOS used an established online panel to recruit 150 participants aged 60 years and older without known pre-existing conditions in Germany. A further 350 patients with pre-existing conditions (see Supplementary Materials, Table S2) were recruited by contacting physicians and support groups. Participation was monetarily reimbursed.

### 2.5. Participant Flow

**Figure 1:**
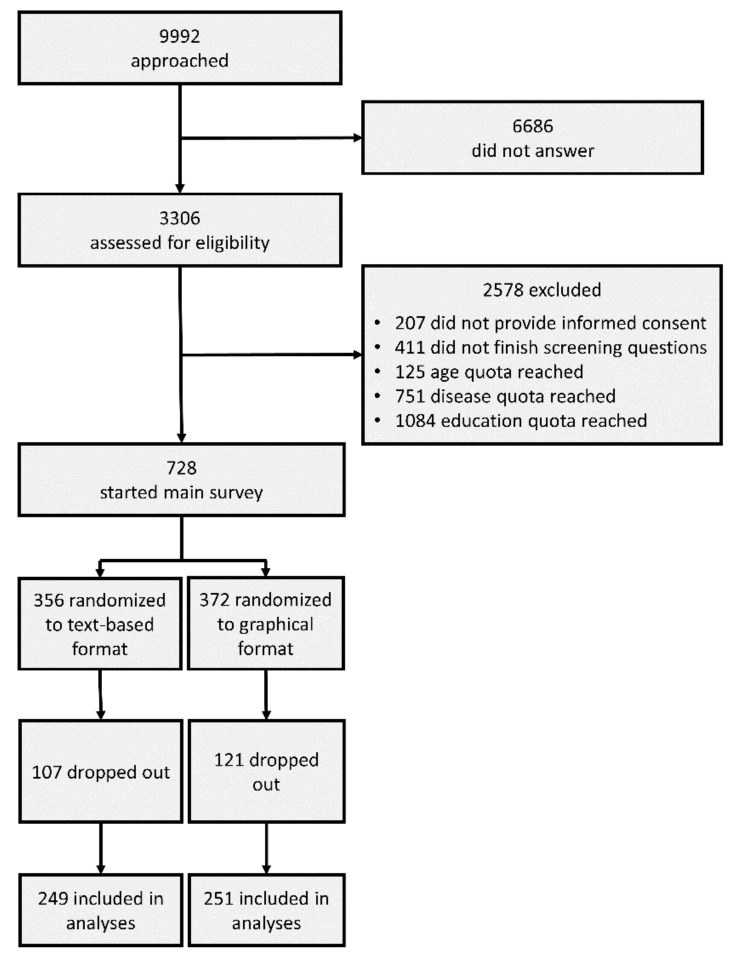
Participant Flow.

IPSOS approached 9992 individuals, of whom 3306 started the trial upon invitation. Of these, 207 did not provide informed consent, 411 abandoned the survey prematurely, and 1960 were rejected because targeted quotas were already filled, leaving 728 participants to start the main survey. Of these, 228 dropped out (text-based: 107; graphical: 121); a final total of 500 participants (text-based: 249, graphical: 251) finished the survey (see Figure 1). Information on gender, age, and education were available for 332 of all participants who dropped out.

### 2.6. Materials

#### Information Formats

**Figure 2:**
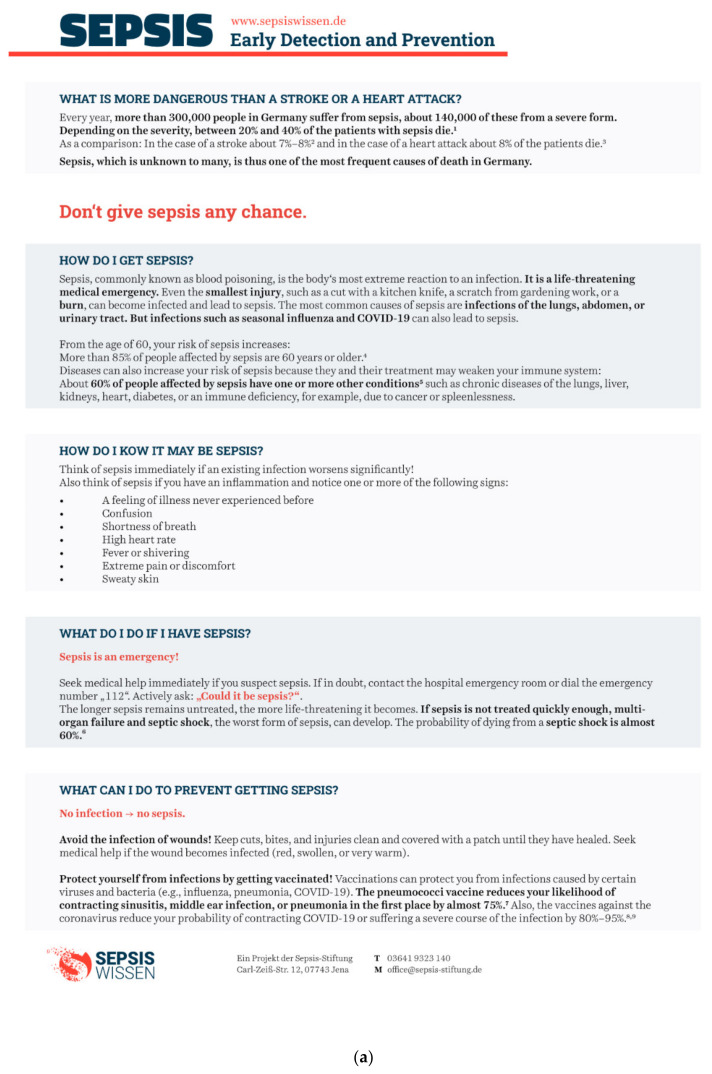

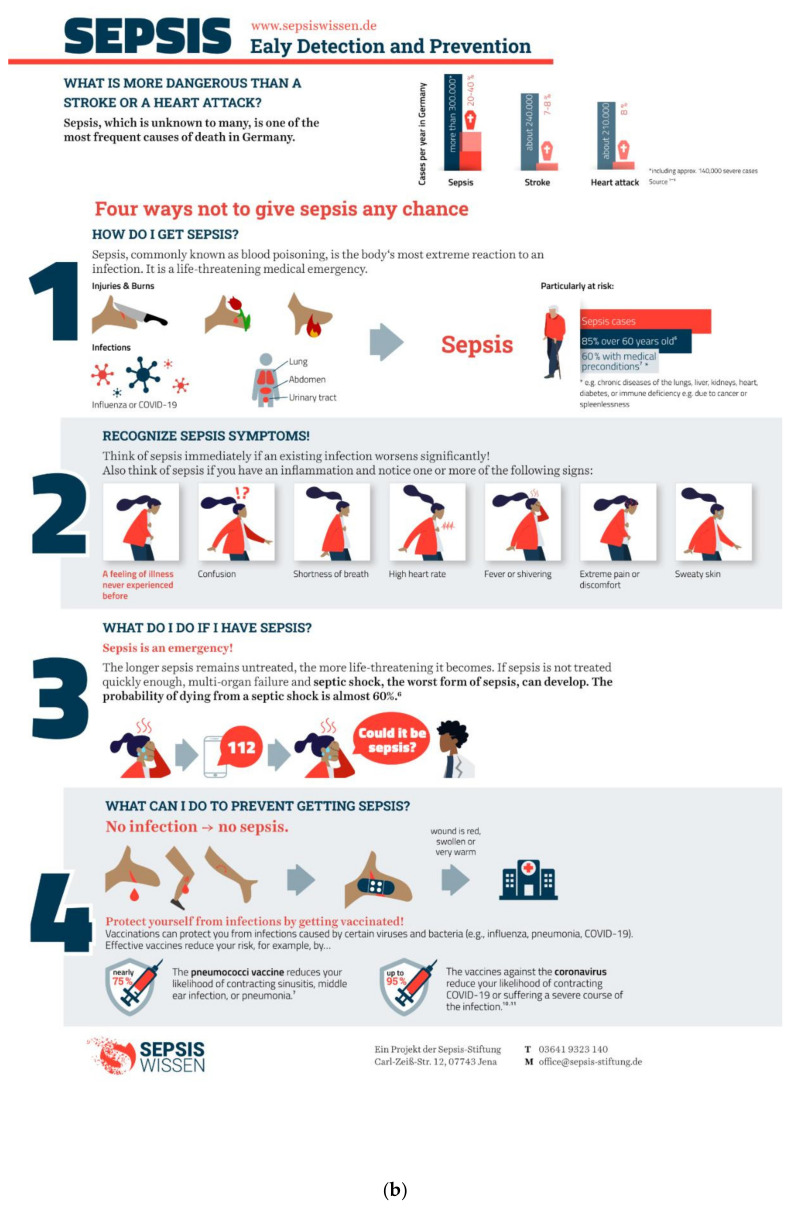
(**a**) English translations of the text-based and (**b**) graphical information formats on prevention and early detection of sepsis.

Based on the guideline for evidence-based health information, the content of the evidence-based formats on prevention and early detection of sepsis was informed by a systematic literature review [41]. Along with general information about the prevalence and mortality of sepsis in Germany (compared with other conditions), the information formats covered three aspects of prevention (wound hygiene, chronic preconditions, and vaccinations) and two aspects of early detection (symptoms and sepsis as an emergency). Comprehensibility was piloted with 10 members of the general public, and the content was revised after feedback. A text-based format and a graphical format of the information were then developed in cooperation with the branding and communications agency Bloominds (Berlin, Germany). The content of the two formats differed only in that the graphical format featured images such as icons and graphs rather than only text. Figure 2 shows the two information formats.

### 2.7. Measures

#### 2.7.1. Informed Choice

The primary endpoint was the validated binary, combined measure of informed choice according to Marteau et al., which differentiates between informed and noninformed choice [10,40]. We separately assessed the prevention and early detection of sepsis. As Marteau’s measure was originally validated on a prenatal screening for Down’s syndrome, it had to be reframed specifically to sepsis for our study (see Supplementary Materials, Figure S1). Three aspects are captured by this measure: risk and health literacy, attitude, and decision.

Risk and health literacy was measured with eight items. For instance, to capture how well participants understood the numerical information provided, they were asked questions such as “How many people do you think get sepsis in Germany every year?” Responses for the item that asked for a count were counted as correct if they were within ±5% of the correct value (i.e., if the right answer was 300,000, responses ranging from 285,000 to 315,000 were counted as correct). Two questions asked for a percentage; answers within ±5 percentage points were counted as correct (e.g., where the right answer was 75%, responses ranging from 70% to 80% were counted as correct). Other questions in this section were aligned to the method used in the European Health Literacy Survey (HLS-EU [42]), which instead of capturing direct knowledge captures people’s impressions of how well they understand, evaluate, and apply health information. Items were adjusted for sepsis and assessed along a four-point Likert scale (e.g., “On a scale from very easy to very difficult, how easy do you find it to remember the most important protective measures against sepsis?”). Health literacy items were counted as adequate if they were rated as very easy or easy. To calculate a participant’s risk and health literacy score, we tallied all their “correct” and “adequate” responses; if they had five or more, they were considered to have sufficient risk and health literacy.

Attitudes toward prevention (“With respect to protection from sepsis, I find vaccinations to be…”) and early detection (“In case of signs for sepsis, being asked to actively draw the attention of physicians to the possibility of sepsis is something that I find to be...”) were assessed with four items, each rated on a four-point Likert scale [38]: reassuring (1)–worrying (4), important (1)–unimportant (4), a good thing (1)–a bad thing (4), an advantage (1)–a disadvantage (4). Attitude was regarded as positive if the mean score across all four options was <2.5.

Decision was assessed with one yes/no question for prevention (“To avoid sepsis, I will have my vaccination status checked promptly and, if necessary, have my vaccinations refreshed”) and another one for early detection (“If I observe any signs of sepsis or a rapid deterioration in my general condition in the future, I will seek medical attention immediately and actively approach the staff about sepsis”).

Informed choice was then combined from the three aspects: A participant’s choice was classified as informed if their risk and health literacy was categorized as adequate and their attitude (positive or negative) corresponded with their final decision. Participants who did not display adequate knowledge or whose attitudes and decision did not match (e.g., who demonstrated a positive attitude toward prevention but decided against it) were classified as making uninformed choices regarding prevention or early detection of sepsis. As a secondary endpoint, we analysed risk and health literacy as its own entity. All endpoints were assessed after participants had seen one or the other information format.

#### 2.7.2. Relevance of Information

To further inform SepsisWissen’s planned sepsis awareness campaign, an additional item asked participants what information about sepsis they consider to be most relevant. Participants could choose 3 pieces of information from a list of 15, including “information about how dangerous sepsis is compared to other diseases” and “the information that a significantly deteriorating infection can indicate sepsis.”

### 2.8. Statistical Methods

Frequencies tested via odds ratios (OR) and chi-square tests were used to analyse differences between the two formats in the primary endpoint of informed choice as well as in sepsis-specific risk and health literacy. In planned subgroup analyses, these differences were also tested within age groups (<60 vs. ≥60 years). Differences between age groups were analysed with chi-square tests. The online questionnaire did not permit item nonresponse; there were therefore no missing values. Data were stored and analysed with IBM SPSS Statistics 26.

## 3. Results

### 3.1. Sample Characteristics

Table 1 reports the distribution of gender, age, and education for participants in the two intervention arms. The distributions of participants exposed to the text-based and graphical formats were similar in terms of gender and age but different in terms of education: There were more participants with higher education in the graphical format group (see Table 1). Details on the diseases of the 350 participants with known pre-existing conditions—including 235 under 60 years of age—are shown in Table 2. All participants without known pre-existing conditions (*n* = 150) were required to be aged 60 or older.

### 3.2. Informed Choice and Risk and Health Literacy for Text-Based and Graphical Formats

Overall, 75% of participants made an informed choice about sepsis prevention after the intervention, and 82% demonstrated adequate risk and health literacy on sepsis prevention. For the early detection of sepsis, 81% made an informed choice after intervention, and 83% exhibited adequate risk and health literacy (see Table 3).

**Figure 3:**
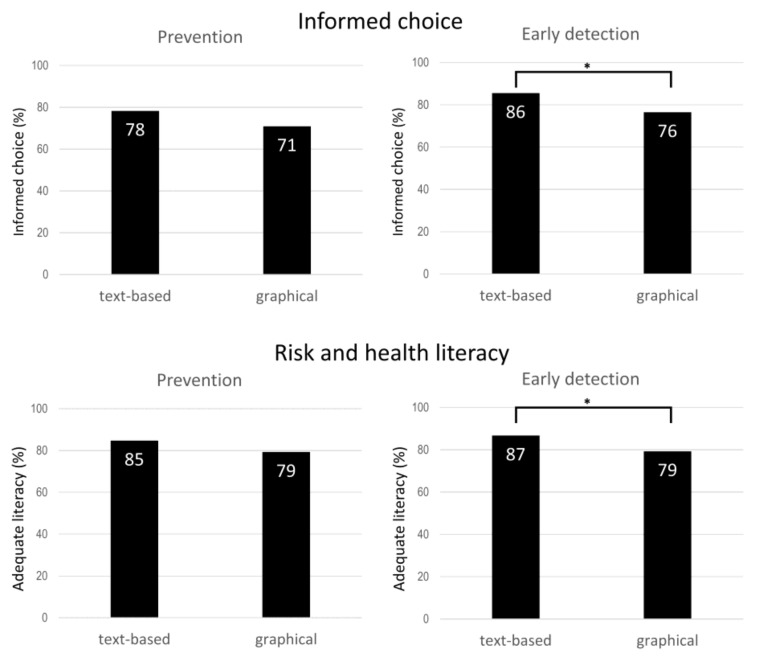
Percentages and statistical significance according to chi-square tests for informed choice and risk and health literacy for text-based and graphical formats. Note: Text-based *n* = 249; graphical *n* = 251. * *p* ≤ 0.05.

The text-based format was associated with higher rates of informed choice (86%) and adequate risk and health literacy (87%) on early detection of sepsis than was the graphical format (informed choice 76%, *p* = 0.012, OR = 1.818; risk and health literacy 79%, *p* = 0.032, OR = 1.710). No such differences between formats were found for sepsis prevention (see Figure 3).

### 3.3. Differences between Age Groups

**Figure 4:**
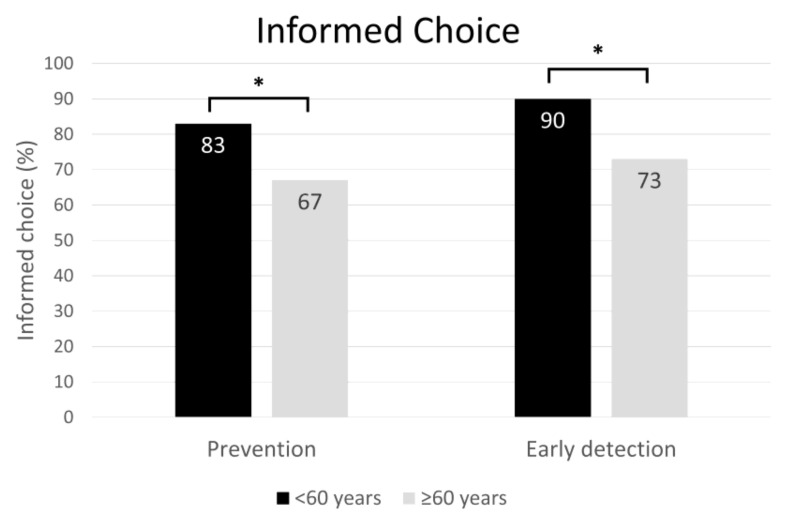
Informed choices and statistical significance according to chi-square test by age group. Note: Under 60 years: *n* = 235; 60 years and older: *n* = 265. * *p* < 0.001.

Of the participants under 60 years old, 83% (prevention) and 90% (early detection) made an informed choice; of participants aged 60 years or older, only 67% (prevention) and 73% (early detection) made an informed choice (see Figure 4; prevention: *p* < 0.001, early detection: *p* < 0.001).

Furthermore, for participants under 60 years old (*n* = 235), the text-based format was associated with more informed choices than was the graphical format for both sepsis prevention (*p* = 0.005, OR = 2.878) and early detection (*p* = 0.027, OR = 1.833). These differences were not found for participants aged 60 years and older (*n* = 265). We did not detect any age-related differences between the two formats in terms of risk and health literacy (see Table 4).

### 3.4. Relevance of Information

**Figure 5:**
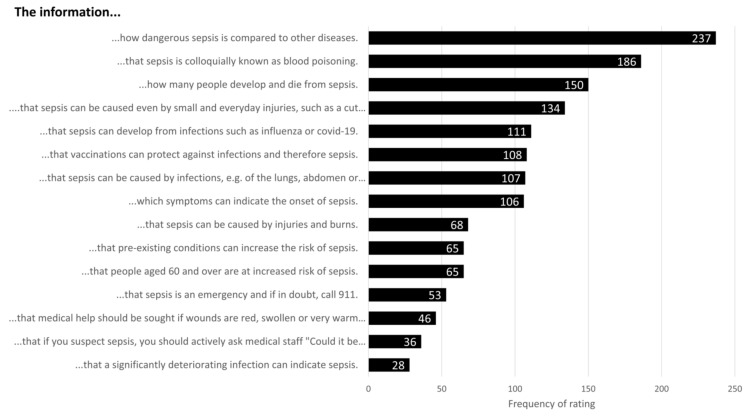
Relevance ratings for sepsis information.

Of 15 pieces of information about sepsis on the list, participants considered 3 to be most relevant. Information about the danger of sepsis in relation to other diseases (e.g., cancer) was rated as relevant most often (237 times), followed by the information that sepsis is colloquially called “blood poisoning” (186 times) and information on the annual incidence and death rate of sepsis (150 times; see Figure 5).

### 3.5. Soft-Launch Data: Pre–Post Differences

Before viewing the information formats, fewer than half of the 30 participants in the soft launch of the study made an informed choice on the early detection (40%) and prevention of sepsis (27%). After seeing the information formats, 63% of participants made an informed choice on early detection and 70% made an informed choice on the prevention of sepsis (see Supplementary Materials, Table S3).

## 4. Discussion

For the prevention and early detection of sepsis, more than 74% of participants made an informed choice after reading either of the health information formats. We found that evidence-based health information in a text-based format was more effective at fostering informed choices and risk and health literacy than a graphical format with regard to the early detection of sepsis; no difference between formats was found for informed choice and risk and health literacy with regard to sepsis prevention. For participants under 60 years old, the text-based format was more effective at fostering informed choices on both the early detection and the prevention of sepsis. These results indicate that low-threshold, easy-to-implement health information has the potential to improve sepsis awareness, particularly in specific risk groups.

Our finding that the text-based format outperformed the graphical format is not completely in line with the findings of Garcia-Retamero and Cokely [36]. In their systematic review of studies on the efficacy of visual aids, they found that 88% of the studies demonstrated visualisations to be more effective than text in promoting risk literacy. However, Garcia-Retamero and Cokely also stressed the importance of numeracy and graph literacy for understanding of visualisations [36]. Because numeracy and graph literacy were not assessed in our study, their effects on our results remains unclear; perhaps a subgroup of participants with at least moderate graph literacy might have profited more from the graphical format than from the text-based format.

Another reason the text-based format performed better in our study might be that the graphical format presented different kinds of visualisations (e.g., bar charts, icons) and contained not only numerical information but also verbal qualitative information. The mix of different components and types of information, as well as the visualisations of complex information (e.g., the course of action for the early detection of sepsis), may have made it harder for participants to grasp the exact meaning of the information in the graphical format than in the text-based format [36]. Although graphical information has been found to be easier to remember, we can only speculate on whether the graphical format would have been better at supporting informed choices in the long run since endpoints were assessed directly after the presentation of the information formats [34,43,44,45]. Future studies with follow-up assessments might shed light on this point.

In the present study, 83% (prevention) and 90% (early detection) of the participants under 60 years old made an informed choice, but only 67% (prevention) and 73% (early detection) of the participants aged 60 years or older did so. Participants younger than 60 years old benefited more from the text-based format than from the graphical format in terms of making an informed choice; this was not the case for participants aged 60 years and older, for whom there was no difference between formats. Although participants under 60 years old differed from participants aged 60 years and older in education, with fewer older participants having received higher education (see Supplementary Materials, Table S4), the results may nevertheless suggest the importance of offering tailored health information formats to address different informational needs. Future studies might for example examine whether individuals aged 60 years and older profit more from other formats, such as experience-based approaches. Information formats such as the single page used in our study could be adapted for print with text-based information on one side and graphical information on the other, thereby addressing the needs of as many people as possible on one sheet of paper.

Another way to increase the relevance—and therefore potentially the effect—of evidence-based health information might be to focus on the information that is especially relevant to the target group. We found that participants tended to regard relatively basic information such as the prevalence and mortality of sepsis, its potential causes, and the fact that sepsis is also known as blood poisoning as most relevant. It might be helpful to centre evidence-based health information on these topics as an engaging starting point.

Our study has limitations that warrant attention. First, since we needed to omit the baseline measure to avoid attentional biases, no pre–post design was employed. Therefore, the present study could only assess the outcomes of evidence-based health information formats after the intervention, and no comparison with the initial state could be made. However, the data from the soft launch, which featured a pre–post design, indicate that both informed choice and risk and health literacy might have been lower before the intervention. This notion is supported by the fact that previous studies have found considerable knowledge gaps regarding sepsis [11,12,13,14]. Future studies should further investigate the effects of evidence-based health information on informed choice and risk and health literacy, for example by implementing a pre–post design with a follow-up assessment. Second, our results are based on a sample with an increased risk of sepsis. Therefore, the results may not generalize to the general population. People with pre-existing conditions or senior citizens might be more sensitive to health-related topics such as sepsis. Moreover, the nonresponse rate was high. We do not know how nonrespondents differed from participants. It is conceivable that participants with stronger interest in the topic had both higher interest in reading the information and higher motivation to complete the entire questionnaire, and that the sample may thus have been selective in that regard. It is also conceivable that participants dropped out because they found the information formats uninformative; in both cases, we might expect that informed choice would have been somewhat lower in participants who dropped out. Nonetheless, comparing participants with people who left the survey prematurely shows that although the former included more men, age and education did not differ, thus indicating that the study sample was not selective in this regard. Third, the components of risk and health literacy were assessed in different ways. While risk literacy was examined using questions in an open numerical or multiple-choice format, health literacy was measured with three items adapted from the HLS-EU, using a Likert scale ranging from “very easy” to “very difficult”. The HLS-EU has been criticized for assessing subjective health literacy rather than health-related knowledge itself [46]. Additionally, some items in the HLS-EU might be answered as “very difficult” even by health professionals and informed individuals since the task itself (e.g., understanding a package leaflet) is very difficult. We chose and adapted the items assessing sepsis specific health literacy carefully to avoid this problem. Third, our study assessed decision as an intention and did not examine whether this intention translated into actual behaviour. Future studies could employ a follow-up measurement to make it possible to assess prevention behaviour (e.g., asking whether participants had had their vaccination status checked), but this might be difficult for early detection because the number of sepsis cases would presumably be low.

## 5. Conclusions

These limitations notwithstanding, our RCT is, to the best of our knowledge, the first study to examine the differences in a text-based and a graphical evidence-based health information format on sepsis regarding informed choice and risk and health literacy. The text-based format was better than the graphical format at fostering informed choice and risk and health literacy for the early detection of sepsis, and younger participants (under 60 years old) seemed to profit more from the text-based than the graphical format in terms of making informed choices, although this was not the case for older respondents (aged 60 years and older). Furthermore, participants regarded information about the prevalence and mortality rates of sepsis, its potential causes, and its colloquial name, “blood poisoning,” as the most relevant information on sepsis. Our findings can form the basis for studies examining the effectiveness of different health information formats for various risk groups and can be used to inform sepsis awareness campaigns on how best to offer evidence-based health information to high-risk target groups.

## Figures and Tables

**Table 1:** Demographic Characteristics of Participants (*n* = 500) in Both Intervention Arms.

	Text-Based Format (*n* = 249)	Graphical Format (*n* = 251)
Age mean (SD)	55.80 (14.03)	56.23 (12.92)
Female	123 (49%)	134 (53%) ^a^
Education		
No formal degree	16 (6%)	8 (3%)
Lower secondary school certificate	77 (31%)	79 (32%)
Secondary school certificate	93 (37%)	104 (41%)
A-levels/technical college entrance qualification	50 (20%)	35 (14%)
University degree	13 (5%)	25 (10%)

Note: Percentages are rounded and may not total 100. ^a^ Two individuals identified as nonbinary and were excluded.

**Table 2:** Pre-existing Conditions of Participants (*n* = 350) in Both Intervention Arms.

Pre-Existing Condition	Frequency	
	Text-Based Format (*n* = 174) (%)	Graphical Format (*n* = 176) (%)
Cancer		
Blood, lymph gland cancer	22 (13%)	17 (10%)
Breast cancer	3 (2%)	7 (4%)
Bowel cancer	9 (5%)	3 (2%)
Urinary bladder, kidney, urinary tract cancer	5 (3%)	5 (3%)
Lung cancer	7 (4%)	6 (3%)
Malignant melanoma of the skin	5 (3%)	5 (3%)
Chronic diseases		
Diabetes mellitus type 1	21 (12%)	15 (9%)
Diabetes mellitus type 2	27 (16%)	25 (14%)
Chronic heart disease	22 (13%)	19 (11%)
Chronic lung disease	20 (11%)	23 (13%)
Chronic renal failure	19 (11%)	17 (10%)
Chronic liver disease	16 (9%)	18 (10%)
Chronic neurological diseases	1 (1%)	3 (2%)
Severe overweight	6 (3%)	5 (3%)
Autoimmune diseases		
Severe rheumatism	18 (10%)	18 (10%)
Severe psoriasis	14 (8%)	14 (8%)
HIV	24 (14%)	32 (18%)
Other autoimmune diseases or diseases with impaired immune function	0	5 (3%)
Therapy that limits immune system function	109 (63%)	102 (58%)

Note: Multiple answers were possible.

**Table 3:** Overall Frequencies for Informed Choice and Risk and Health Literacy for Prevention and Early Detection of Sepsis.

	Informed Choice		Risk and Health Literacy	
	Prevention	Early Detection	Prevention	Early Detection
Uninformed/inadequate *n* (%)	127 (25%)	95 (19%)	90 (18%)	85 (17%)
Informed/adequate *n* (%)	373 (75%)	405 (81%)	410 (82%)	415 (83%)

**Table 4:** Frequencies and statistical significance in chi-square test for informed choice and risk and health literacy by age group.

		Informed Choice						Risk and Health Literacy					
		Prevention			Early Detection			Prevention			Early Detection		
		Uninformed	Informed	*p*	Uninformed	Informed	*p*	Inadequate	Adequate	*p*	Inadequate	Adequate	*p*
<60 years (*n* = 235)	text-based (n = 115)	11	104	0.005 *	6	109	0.027 *	4	111	0.108	5	110	0.054
	graphical (n = 120)	28	92		17	103		11	109		14	106	
≥60 years (*n* = 265)	text-based (n = 134)	43	91	0.794	30	104	0.097	34	100	0.340	28	106	0.155
	graphical (n = 131)	45	86		42	89		41	90		38	93	

Note: * *p* ≤ 0.05.

## Data Availability

The data presented in this study are openly available in OSF (doi 10.17605/OSF.IO/85DHZ).

## Supplementary Materials

The following supporting information can be downloaded at: https://www.mdpi.com/article/10.3390/jcm11133659/s1, Table S1: Pre-set criteria for the recruitment of participants with and without pre-existing conditions and actual frequencies in the sample; Table S2: Demographic characteristics of participants with and without pre-existing conditions; Figure S1: English translation of the survey items on informed choice; Table S3: Frequencies and percentages for informed choice and for adequate risk-and-health literacy for the prevention and early detection of sepsis for the 30 participants of the soft-launch with a pre-post design; Table S4: Demographic characteristics of participants under and those ages 60 years and older.
