# Supplementary material for: Text-Based vs. Graphical Information Formats in Sepsis Prevention and Early Detection: A Randomized Controlled Trial on Informed Choice
Source: J Clin Med. 2022 Jun 24;11(13):3659. doi: 10.3390/jcm11133659 (PMC9267388; doi:10.3390/jcm11133659)
Supplement: Supplementary file 1 [file jcm-11-03659-s001.zip › Supplementary Materials.pdf]

Supplementary Materials

Table S1. Pre-set criteria for the recruitment of participants with and without pre-existing conditions and actual frequencies in the sample.

Pre-existing condition	Pre-set rates	Frequencies
Cancer		
Blood, lymph gland cancer	35	55
Breast cancer	7	10
Bowel cancer	7	12
Urinary bladder, kidney, urinary tract cancer	7	10
Lung cancer	7	13
Malignant melanoma of the skin	7	10
Chronic diseases		
Diabetes mellitus type 1	26	36
Diabetes mellitus type 2	27	52
Chronic heart disease	35	41
Chronic lung disease	35	43
Chronic renal failure	26	36
Chronic liver disease	26	34
Chronic neurological diseases	0	4
Severe overweight	0	11
Autoimmune diseases		
Severe rheumatism	26	36
Severe psoriasis	26	28
HIV	53	56
Other autoimmune diseases or diseases with impaired immune function	0	5

Table S2. Demographic characteristics of participants with and without pre-existing conditions.

	Participants without pre-existing conditions (n=150) %	Participants with pre-existing conditions (n=350) %
Female	38	57
Age (in years)		
<20	0	0
20–39	0	19
40–59	0	48
60–79	99	33
≥80	1	0
Education		
No formal degree	0	7
Lower secondary school certificate	49	23
Secondary school certificate	28	44
A-levels/ technical college entrance qualification	12	19
Graduation from university	11	6

Note: Percentages are rounded and may not total 100.

Figure S1. English translation of the survey items on informed choice.

PREVENTION

Risk-and-health literacy (the first three items are identical for prevention and early detection)

1. How many people do you think get sepsis in Germany every year?
   approx. _ _ _ people *(<300.000)*

2. What percentage of people suffering from sepsis in Germany die?
   approx. _ _ _ % *(20-40%)*

3. What can increase the risk of sepsis?
   - Insufficient fluid intake
   - Age between 20 and 60 years
   - Veganism (a purely plant-based diet)
   - *Chronic diseases such as diabetes, lung diseases, cancer, kidney diseases*

4. What do you think can be done to prevent sepsis from developing?
   - *Good wound hygiene and certain vaccinations can protect against infections and sepsis.*
   - Sepsis cannot be prevented.
   - Drinking enough can protect you from sepsis.
   - Frequent consumption of processed foods should be avoided to prevent sepsis.

5. How effective is pneumococcal vaccination in preventing illnesses such as sinusitis, middle ear infection or pneumonia, which can lead to sepsis?
   approx. _ _ _ % *(75%)*

6. On a scale from "very easy" to "very difficult" how easy do you find it ...

	very easy	easy	difficult	very difficult
... to remember the most important protective measures against sepsis?	○	○	○	○
... to assess which steps are the most appropriate to prevent the development of sepsis?	○	○	○	○
... to make a decision on how you should protect yourself against sepsis?	○	○	○	○

Attitude

7. “With respect to protection from sepsis, I find vaccinations to be…

	1	2	3	4	
reassuring	○	○	○	○	worrying
important	○	○	○	○	unimportant
good thing	○	○	○	○	bad thing
an advantage	○	○	○	○	a disadvantage

Decision

8. To avoid sepsis, I will have my vaccination status checked promptly and, if necessary, have my vaccinations refreshed.
   - Yes
   - No

EARLY DETECTION

Risk-and-health literacy (the first three items are identical for prevention and early detection)

1. How many people do you think get sepsis in Germany every year?
   approx. _ _ _ people *(<300.000)*

2. What percentage of people suffering from sepsis in Germany die?
   approx. _ _ _ % *(20-40%)*

3. What can increase the risk of sepsis?
   - Insufficient fluid intake
   - Age between 20 and 60 years
   - Veganism (a purely plant-based diet)
   - *Chronic diseases such as diabetes, lung diseases, cancer, kidney diseases*

4. What are typical signs of sepsis?
   - Confusion, stomach pain, weakness in arm and leg.
   - *Unprecedented feeling of illness, confusion, fever or trembling.*
   - Sweaty skin, nosebleeds, red eyes.
   - Itchy scalp, difficulty swallowing, loss of appetite.

5. What should you do if you have signs of sepsis?
   - Wait a day and then decide.
   - *Seek medical help and actively ask: "Could it be sepsis?", if necessary, contact the emergency room or dial "112".*
   - As long as there is no red line on the wound, nothing needs to be done.
   - Get some rest and recover at home.

6. On a scale from "very easy" to "very difficult" how easy do you find it ...

	very easy	easy	difficult	very difficult
... to remember the most important signs that indicate sepsis?	○	○	○	○
... to assess whether signs of illness could indicate sepsis?	○	○	○	○
... to decide what to do when you notice signs of sepsis?	○	○	○	○

Attitude

7. In case of signs for sepsis, being asked to actively draw the attention of physicians to the possibility of sepsis, is something that I find to be...

	1	2	3	4	
reassuring	○	○	○	○	worrying
important	○	○	○	○	unimportant
good thing	○	○	○	○	bad thing
an advantage	○	○	○	○	a disadvantage

Decision

8. If I observe any signs of sepsis or a rapid deterioration in my general condition in the future, I will seek medical attention immediately and actively approach the staff about sepsis.
   - Yes
   - No

Table S3. Frequencies and percentages for informed choice and for adequate risk-and-health literacy for the prevention and early detection of sepsis for the 30 participants of the soft-launch with a pre-post design.

	Informed choice		Risk-and-health literacy	
	**Pre**	**Post**	**Pre**	**Post**
Early Detection *n* (%)	12 (40%)	19 (63%)	12 (40%)	19 (63%)
Prevention *n* (%)	8 (27%)	21 (70%)	10 (33%)	23 (77%)

Table S4. Demographic characteristics of participants under and those ages 60 years and older.

	Participants < 60 years (n=235) %	Participants ≥ 60 years (n=265) %
Female	57	46
Education		
No formal degree	7	3
Lower secondary school certificate	17	44
Secondary school certificate	44	36
A-levels/ technical college entrance qualification	24	11
Graduation from university	9	7

Note: Percentages are rounded and may not total 100.
